# Excess weight in children living in rural areas related to the nutritional profile and to maternal habits

**DOI:** 10.17533/udea.iee.v39n1e03

**Published:** 2021-03-05

**Authors:** Erika Barbosa Lagares, Paulo Henrique Alves Sousa, Karolyne Araujo Resende, Letícia Camilo Santos, Luiz Henrique Rodrigues Silva, Vinícius Silva Belo, Márcia Christina Caetano Romano

**Affiliations:** 1 Nutritionist, MS. Federal University of São João Del-Rei- UFSJ-CCO, Divinópolis-MG, Brazil. Email: erikablagares@yahoo.com.br Universidade Federal de São João del-Rei Federal University of São João Del-Rei DivinópolisMG Brazil erikablagares@yahoo.com.br; 2 Nutritionist, MS student. Federal University of São João Del-Rei- UFSJ-CCO, Divinópolis-MG, Brazil. Email: spaulohenrique@hotmail.com Universidade Federal de São João del-Rei Federal University of São João Del-Rei DivinópolisMG Brazil spaulohenrique@hotmail.com; 3 Nurse, MS. Federal University of São João Del-Rei- UFSJ-CCO, Divinópolis-MG, Brazil. Email: karolyne.resende@gmail.com Universidade Federal de São João del-Rei Federal University of São João Del-Rei DivinópolisMG Brazil karolyne.resende@gmail.com; 4 Undergraduate Nursing student. Federal University of São João Del-Rei- UFSJ-CCO, Divinópolis-MG, Brazil. E-mail: leticiacamilosantos2012@gmail.com Universidade Federal de São João del-Rei Federal University of São João Del-Rei DivinópolisMG Brazil leticiacamilosantos2012@gmail.com; 5 IT technician. Federal Center of Technological Education of Minas Gerais (Centro Federal de Educação Tecnológica de Minas Gerais, CEFET-MG), Divinópolis-MG, Brazil. E-mail: luizhrsilva.2000@gmail.com Centro Federal de Educação Tecnológica de Minas Gerais Federal Center of Technological Education of Minas Gerais DivinópolisMG Brazil luizhrsilva.2000@gmail.com; 6 Biologist, PhD. Professor. Federal University of São João Del-Rei- UFSJ-CCO, Divinópolis-MG, Brazil. Email: viniciusbelo4@hotmail.com Universidade Federal de São João del-Rei Federal University of São João Del-Rei DivinópolisMG Brazil viniciusbelo4@hotmail.com; 7 Nurse, PhD. Professor. Federal University of São João Del-Rei- UFSJ-CCO, Divinópolis-MG, Brazil. Email: marciachristinacs@gmail.com Universidade Federal de São João del-Rei Federal University of São João Del-Rei DivinópolisMG Brazil marciachristinacs@gmail.com

**Keywords:** child, obesity, nutritional status, feeding behavior, mother-child relations, niño, obesidad, estado nutricional, conducta alimentaria, relaciones madre-hijo., criança, obesidade, estado nutricional, comportamento alimentar, relações mãe-filho

## Abstract

**Objective.:**

To assess the relationship between the nutritional status and eating habits of children aged from five to ten years old and their mothers, living in rural areas.

**Methods.:**

A cross-sectional study conducted with 156 children aged from five to ten years old, registered in the Family Health Strategies of the rural area of the Municipality of Divinópolis-MG (Brazil) from July 2017 to April 2018.

**Results.:**

The prevalence of excess weight was 27.5%. The following parameters were significantly associated with excess weight in the children: maternal waist circumference (OR=1.04), protein consumption (OR=1.02), irregular consumption of natural juice (OR=5.05), and the most favored socioeconomic level, C1 social stratum (OR=3.54). Regarding the correlation between nutrient intake of the children and their mothers, most of the correlations were weak to moderate, being statistically significant for all the dietary components evaluated (r=0.185 to 0.496).

**Conclusion.:**

Maternal nutritional status was related to the child's excess weight and a weak to moderate correlation was observed for nutrient intake among the children and their mothers. A high prevalence of children with excess weight was observed in the rural areas. The results point to the need to implement collective approaches, targeted at rural families, so as to prevent this problem.

## Resumen

**Objetivo.:**

Evaluar la relación entre el estado nutricional y los hábitos alimentarios de los niños de cinco a diez años y sus respectivas madres, las cuales residen en zonas rurales.

**Métodos.:**

Se trató de un estudio transversal realizado con 156 niños de cinco a diez años, registrados en las Estrategias de Salud de la Familia del área rural del municipio de Divinópolis-MG (Brasil) de julio de 2017 a abril de 2018.

**Resultados.:**

La prevalencia de sobrepeso fue del 27.5%. Se asoció significativamente con el sobrepeso de los niños: la circunferencia de la cintura materna (OR=1.04), el consumo de proteínas (OR=1.02), el consumo irregular de jugo natural (OR=5.05) y el nivel socioeconómico más favorecido, estrato social C1 (OR=3.54). En cuanto a la correlación entre la ingesta de nutrientes de los niños y la de sus madres, la mayoría de las correlaciones fueron débiles o moderadas, siendo estadísticamente significativas para todos los componentes dietéticos evaluados (r=0.185 a 0.496).

**Conclusión.:**

El estado nutricional de la madre se relacionó con el sobrepeso del niño y se observó una correlación de débil a moderada para la ingesta de nutrientes entre los niños y sus madres. Se identificó una alta prevalencia de niños con sobrepeso en las zonas rurales. Los resultados apuntan a la necesidad de implementar enfoques colectivos, dirigidos a familias del medio rural, para prevenir este problema.

## Resumo

**Objetivo.:**

Avaliar a relação entre estado nutricional e hábitos alimentares de crianças de cinco a dez anos e de suas respectivas mães, residentes em zona rural.

**Métodos.:**

Estudo transversal, realizado com 156 crianças de cinco a dez anos, cadastradas nas Estratégias de Saúde da Família da zona rural do município de Divinópolis-MG (Brasil), de julho de 2017 a abril de 2018.

**Resultados.:**

A prevalência de excesso de peso foi 27.5%. Associou-se significativamente ao excesso de peso das crianças: a circunferência de cintura materna (OR=1.04), o consumo de proteína (OR:1.02), o consumo irregular de suco natural (OR=5.05); o nível socioeconômico mais favorecido, estrato social C1 (OR=3.54). No que diz respeito à correlação entre o consumo de nutrientes das crianças e suas mães, a maioria das correlações foi de fraca a moderada, sendo significativas estatisticamente para todos os componentes dietéticos avaliados (r=0.185 a 0.496).

**Conclusão.:**

O estado nutricional materno relacionou-se ao excesso de peso da criança e observou-se correlação fraca-moderada para ingestão de nutrientes entre as crianças e suas mães. Foi identificada elevada prevalência de excesso de peso infantil na zona rural. Os resultados apontam a necessidade de implementação de abordagens de ordem coletiva, voltadas às famílias de áreas rurais, para prevenção desse agravo.

## Introduction

Obesity constitutes an important public health problem. The prevalence of excess weight in children rose from 16.9% in 1980 to 23.8% in 2013 in boys and from 16.2% to 22.6% in girls, especially in developed countries. In developing countries, the prevalence of excess weight rose from 8.1% in 1980 to 12.9% in 2013 in boys and from 8.4% to 13.4% in girls.(1) In Brazil, between 1974-1975 and 2008-2009, the prevalence of excess weight in children from five to nine years old rose from 10.9% to 34.8% in the male gender and from 8.6% to 32.0% in the female gender. The trend was also increasing for obesity in those years, rising from 2.9% to 16.6% in boys and from 1.8% to 11.8% in girls. The increase in excess weight from the age of five was detected in all income groups and regions of the country.(2)

When comparing the evolution of the median weight of children under nine years of age depending on the residence area (urban or rural), there is a tendency for those in rural areas to have a nutritional aspect similar to those in urban areas, since the evolution curve of the median weight of these children is almost overlapped with the curve of the expected pattern.(2) This suggests that the nutritional transition also affects the rural area, and that, despite the fact that the family's work practices in the countryside involve planting and harvesting, which would favor physical exercise and access to *in natura* food, there is an increase in consumption of processed products and sedentary behaviors among children and their families.(3) The situation is worrying, since obese children are more likely to become obese adolescents and adults, and may consequently present associated chronic diseases,(4) which will increase public spending on health, in addition to reducing time and quality of life.(5)

It is believed that the formation of eating habits has a great contribution from the family, since children, besides having their eating preferences genetically influenced, tend to imitate the preferences, attitudes, beliefs and behaviors of their parents.(6) However, when investigating the family influence on child excess weight, the most prominent role seems to be that of the mother, who is often the primary caregiver.(7) Possibly, these elements contribute to the rise of excess weight in children, also in rural environments(8) and, considering all the implications of childhood obesity, it is relevant to assess this context.

It is worth mentioning that, both in Brazil and worldwide, there are few manuscripts that assess in greater depth and simultaneously the relationship between eating habits and nutritional status of children and their mothers, and most of the existing studies are international and include only urban environments. Therefore, there is a gap in studies that address this issue in rural residents. It is imperative for Nursing to have a greater understanding of the theme, since it directly acts in monitoring the child's growth and development, and can contribute to health promotion through guidelines on appropriate eating habits.(9) In view of this, the present research aims to assess the relationship between nutritional status and eating habits of children and their mothers, living in rural areas.

## Methods

This is a qualitative study of a cross-sectional nature. The research was conducted along with four Family Health Strategy (FHS) units located in the rural area of the Municipality of Divinópolis, state of Minas Gerais, Brazil. The FHS consists in a basic care assistance model, based on the work of a multi-professional team in an attached territory, and performs health actions triggered by the local reality and by the needs of the population covered. This model favors the closeness of the health unit to the families, promotes access to the services, and facilitates the establishment of bonds between the team and the users, which results in greater impact on the local health condition.(10)

The study population consisted of 307 children aged between five and ten years old, registered in the FHS units in rural areas in 2017, and their respective mothers. The sample size was calculated using the Open Epi program, version 3.01, for a confidence level of 95%, accuracy of 5% and proportion of excess weight in the municipality of 28.85%, according to a report by SISVAN-WEB,(11) which led to an estimated sample of 156 children. Stratified sampling was carried out, considering four allocation strata represented by the FHS teams in the rural area, with the participants being selected in a simple random way.

The following inclusion criteria were adopted: children aged between five and ten years old and their mothers attended by the FHS units in the rural area of the Municipality of Divinópolis. The following exclusion criteria were considered: children with some illness, genetic disease or syndrome, which could add bias to the research. Data collection took place in the period from July 2017 to April 2018. The socioeconomic level of the families was assessed using the questionnaire and classification criteria of the Brazilian Association of Research Companies (*Associação Brasileira de Empresas de Pesquisa*, ABEP).(12) Dietary intake was evaluated by the 24-hour food reminder survey for both children and mothers. The foods were quantified in calories and nutrients with the aid of the Avanutri® software, version 4.0.

The anthropometric assessment (weight, height, waist and arm circumference of children and their mothers) was performed according to technical procedures recommended by the WHO and the Brazilian Ministry of Health.(13) The measurements were performed in triplicate and the arithmetic mean of the data obtained was then calculated. The current weight of the mother and child was obtained by measuring on a Tanitta® digital scale, model HD-313, with 100g precision and a maximum capacity of 150 kilograms (kg). The children and their mothers were barefoot and in light clothes, positioned on the scale with their feet together, body erect and looking at the horizon, so that their body weight was evenly distributed on both feet. After stabilization of the measurement presented by the scale, weight was recorded.(13)

The portable Alturexata® stadiometer, with a vertical mobile rod with a scale in centimeters (cm) and a variation of one millimeter (mm), was used to measure height. The children and mothers evaluated stood with their backs to the equipment, barefoot, with their feet together, body erect, looking towards the horizon, and with their arms relaxed along their bodies. The mobile part of the stadiometer was placed at the highest point of the head, so that the height reading could be performed.(13)

The waist circumference of the child and the mother were measured with an inelastic measuring tape, during normal expiration, with the midpoint between the margin of the last rib and the iliac crest as the reference point.(14) Anatomical markings to measure the arm circumference, of both mother and child, were performed with the arm flexed towards the chest, forming a 90° angle. The midpoint between the acromion and the olecranon was identified and marked. The arm was contoured with the inelastic measuring tape at the marked point, in an adjusted way, avoiding skin compression or slack. Measurement reading was performed with the arm along the body.(15)

For the nutritional assessment of the children, the *WHO Anthro Plus* program of the World Health Organization (WHO) was used, establishing as evaluation index the Body Mass Index (BMI) by age according to the WHO curves. BMI calculation was also used, classifying them according to the WHO criteria, for the nutritional assessment of the mothers.(13)

The dependent variable (outcome) was excess weight in the child. The children with excess weight were those classified as overweight, obesity, and severe obesity. The independent (explanatory) variables were selected considering demographic characteristics (gender, age, skin color), socioeconomic status,(12) clinical data (emotional characteristics, sleep time, habit of eating in front of the TV, screen time), dietary intake (energy, macro- and micro-nutrients), maternal characteristics and habits (BMI, anthropometry, dietary intake). The data collected were processed in the Epidata® program, version 3.1, by means of double entry, which allowed for due data consistency and validation analysis. Data analysis was performed in the Statistical Package for Social Sciences (SPSS Inc., Chicago, IL) software, version 20.0, from the calculation of the distributions of frequencies and measures of central tendency and dispersion. Pearson's chi-square test/Fisher's Exact test or the Mann-Whitney's test were used, respectively, for comparing the percentages and medians.

The Spearman's Correlation test was used to analyze the dietary habits of children and their mothers for the quantitative variables of food consumption. The strength of the correlations was considered weak when the coefficients were below 0.30; moderate, when the *r*values were between 0.30 and 0.50; and strong, when r ≥ 0.50.(16) In addition, multivariate logistic regression models were built using non-automatic methods of retroactive selection of variables to control confounding between the variables associated with excess weight. A significance level of 5% was used.

The research project was approved by the Ethics and Research Committee of the Federal University of São João Del-Rei (*Universidade Federal de São João Del-Rei*, UFSJ) under opinion number: 1,945,317 and CAAE: 62370816.2.0000.5545. A free and informed consent form was given to the population studied, with information regarding the objectives of the research, their rights, as well as the risks and benefits arising from the research, assuring them of the anonymous nature of the interviewees and the freedom to refuse to participate or withdraw from the research at any moment.

## Results

A total of 156 pairs of children and their respective mothers were evaluated. Considering that among the drawn children were siblings, the number of mothers totaled 138. The children presented a median of 7.5 years old, varying between 5 and 9.9 years old; 55.8% were male; and 27.5% had excess weight. The participants presented a median of 10 sleep hours a day, always ate in front of the TV (64.1%) and were considered calm (42.9%) and agitated (34.6%) (Table 1).

**Table 1:** Socioeconomic and health characteristics of the 156 children of the study

Variables	Value
Sociodemographic characteristics	
**Age group; *n*(%)**	
5 years old	24 (15.4)
6 years old	29 (18.6)
7 years old	25 (16)
8 years old	40 (25.6)
9 years old	38 (24.4)
**Gender; *n*(%)**	
Female	69 (44.2)
Male	87 (55.8)
**Skin color; *n*(%)**	
White	86 (55.1)
Brown	60 (38.5)
Black	10 (6.4)
Lifestyle characteristics	
Sleep hours; median (min-max)	10.0 (7.0-12.5)
Screen Time; *n*(%)	
≤ 2 hours a day	63 (40.4)
> 2 hours a day	93 (59.6)
**Eats in front of the TV; *n*(%)**	
Always	100 (64.1)
Sometimes	26 (16.7)
Never	30 (19.2)
**Emotional temper; *n*(%)**	
Calm	67 (42.9)
Agitated	54 (34.6)
Anxious	35 (22.4)
Anthropometric characteristics	
Nutritional state; *n*(%)	
Thinness	5 (3.2)
Eutrophy	108 (69.2)
Overweight	28 (17.9)
Obesity	15 (9.6)
Severe Obesity	0 (0.0)
Waist circumference (cm); median (min-max)	55.7 (41.7-93.0)
Arm circumference (cm); median (min-max)	18.5 (13.5-32.5)
Waist/Height ratio; median (min-max)	0.44 (0.36-0.61)

The children's mothers had a mean age of 34.9 years old, 60.1% presented excess weight, 3.6% had diabetes mellitus, 11.6% had arterial hypertension, and 8.0% had thyroid disorder. Regarding the socioeconomic level, 44.2% of the families were grouped in class D-E, which represents the least favored social class, and 5.4% are beneficiaries of the government income transfer program called *Bolsa Família*.

Regarding the correlation between the consumption of nutrients by children and their mothers, most of the correlations were weak to moderate, being statistically significant for all the dietary components evaluated considering the analysis without stratification (r=0.185 to 0.496).(16) The correlations were slightly stronger among children with excess weight only for proteins, polyunsaturated fats, vitamin A, vitamin E and zinc (Table 2).

**Table 2:** Spearman's correlation coefficient between the dietary intake of 156 children and their mothers according to the presence of excess weight in the child and to gender

Dietary Intake		Gender (child)	Gender (child)	**Excess Weight** **(child)**	**Excess Weight** **(child)**
(Energy, macro- and micro-nutrients)	Total	Fem.	Male	No	Yes
Energy (kcal)	0.299^*^	0.272^*^	0.324^*^	0.367^*^	0.183
Protein (% TCV)	0.346^*^	0.389^*^	0.309^*^	0.322^*^	0.428^*^
Carbohydrate (% TCV)	0.251^*^	0.289^*^	0.222^*^	0.247^*^	0.250
Lipid (% TCV)	0.385^*^	0.478^*^	0.303^*^	0.392^*^	0.341^*^
Cholesterol (mg)	0.185^*^	0.102	0.261^*^	0.205^*^	0.108
Saturated Fat (g)	0.313^*^	0.250^*^	0.360^*^	0.410^*^	0.129
Polyunsaturated Fat (g)	0.316^*^	0.257^*^	0.355^*^	0.252^*^	0.439^*^
Monounsaturated Fat (g)	0.404^*^	0.475^*^	0.357^*^	0.456^*^	0.313^*^
Fibers (g)	0.324^*^	0.343^*^	0.317^*^	0.391^*^	0.203
Vitamin A (Re)	0.410^*^	0.475^*^	0.363^*^	0.384^*^	0.424^*^
Vitamin D (mcg)	0.331^*^	0.301^*^	0.359^*^	0.377^*^	0.198
Vitamin E (mg)	0.296^*^	0.312^*^	0.297^*^	0.251^*^	0.408^*^
Calcium (mcg)	0.330^*^	0.262^*^	0.380^*^	0.350^*^	0.246
Iron (mg)	0.299^*^	0.246^*^	0.342^*^	0.321^*^	0.288
Zinc (mg)	0.496^*^	0.491^*^	0.494^*^	0.486^*^	0.573^*^

* p-value <0.05. % TCV: Percentage of the Total Caloric Value.

In the analysis of multivariate association between the explanatory variables and the excess weight of the children we have the following: maternal waist circumference (OR=1.04), protein consumption (OR=1.02), irregular consumption of natural juice (OR=5.05), and CI social stratum socioeconomic level (OR=3.54) (Table 3).

**Table 3:** Variables associated with excess weight in the 156 children from 5 to 10 years old, living in rural areas, according to the multivariate regression model

Associations with excess weight in children	***p*-value**	OR	CI (95%)
Variables of the child			
Protein consumption (g)	0.001	1.02	(1.01 - 1.04)
Irregular Consumption of Natural Juice (< 5 times a week)	<0.001	5.05	(2.09 - 12.2)
Socioeconomic level - ABEP			
B2	0.134	4.91	(0.61 - 39.46)
C1	0.021	3.54	(1.21 - 10.38)
C2	0.950	0.97	(0.34 - 2.73)
D-E*	0.046	1	
Maternal variable			
Waist Circumference (cm)	0.023	1.04	(1.01 - 1.07)

(*) Reference stratum for associations of the socioeconomic level (lowest level).

## Discussion

In the present study, children's excess weight was significantly related to the maternal nutritional status, observed by the direct association with the measurement of the mother's waist circumference, which increased by 0.04 times the chance of the child having excess weight. Corroborating this result, a Brazilian study carried out with children aged three to ten years old observed that a mother with accumulation of abdominal fat increased by 2.7 times the chance of the child also presenting this condition.(17) This indicates that the maternal nutritional status can also be related to the outcome of childhood obesity in rural areas. It must be considered that such influence can be associated with both genetic and sociocultural factors of family habits. Therefore, it is essential that the care with nutritional attention to maternal-infant health begins in the prenatal period, considering the entire family structure.(17)

The socioeconomic level was significantly associated with excess weight in the children, being that children allocated to level C1, had a 3.54-fold chance of presenting this problem when compared to children at level D-E, who represent the less favored class. In fact, greater purchasing power also favors access to a nutritionally inadequate dietary pattern, such as consumption of sugary drinks, sweets and high energy density industrialized foods.(18) In addition, a review that included studies published between 1985 and 2008 pointed out that this outcome can differ between geographic regions, since residents of rural areas are more affected by precarious access to supermarkets and healthy foods.(19)

The child's protein intake presented a 1.02 chance ratio for excess weight. A similar result was found in a population-based longitudinal study carried out in Portugal, which assessed the nutritional status outcome of children aged four years old at baseline after three years, and found that protein consumption was significantly associated with a higher BMI both in boys and girls.(20) This phenomenon is justified by the fact that excessive protein intake can stimulate insulin secretion as well as IGF 1, which is a growth factor similar to insulin, and both contribute to increased adipogenesis and differentiation of adipocytes, thus inducing obesity.(21)

In this research it was observed that children who reported irregular consumption (less than five times a week) of natural fruit juice had a 5.05 chance of presenting excess weight. Corroborating this finding, a Canadian survey indicated that 56.8% of the respondents reported consuming fruit juice regularly. These authors suggested that regular consumption of natural fruit juice would be associated with eutrophy.(22) These results are possibly explained by the fact that the habit of drinking natural juice regularly causes the child to stop drinking other sugary drinks, with higher energy density and without significant nutritional values, such as box juice, soft drinks and soda. It is worth noting that the high consumption of sugary non-alcoholic beverages has been systematically discussed in the literature as having a possible positive association with the BMI of children and adolescents.(23)

The present study identified a prevalence of 27.5% of excess weight among children living in rural areas, considered higher than the results of another Brazilian study of national scope.(2) A systematic literature review identified, among nine articles included, three international studies that found a higher prevalence of childhood obesity in the rural area when compared to the urban area. The findings of these studies are justified by the food intake poor in nutrients and excessive in fats, in addition to the lower practice of physical exercise and use of electronic equipment during longer periods by children in the urban area.(24)

The study has limitations, including the application of the 24-hour food reminder, which, for being a survey from the previous day, may not represent the individual's usual intake. However, despite its limitations, the 24-hour reminder is still the most appropriate method for estimating the food consumption of a population.(25) The research design allowed us to estimate the prevalence of excess weight in children living in the rural area of the Municipality of Divinópolis, as well as its association with maternal habits and nutritional status, not being possible, due to the cross-sectional nature, to make cause and effect inferences. However, this design answered the research question and the study objectives.

The findings of this research bring potential contributions to the Brazilian literature, since it makes important clarifications about factors associated with excess weight in children from rural areas. The association of the children's excess weight with the mother's waist circumference stands out, which points to the lack of interventions, especially in family planning of residents in rural areas, in order to provide guidance on the importance of the maternal nutritional status, habits and lifestyle for the health of their children, thereby reducing the need for treatment of the comorbidities resulting from childhood obesity. However, this result must be analyzed with caution, as more research studies must be carried out in rural areas, including longitudinal ones, for any generalization.

The results of this study allow us to conclude that the child's excess weight is related to the maternal nutritional status, evidenced especially by the direct association between the measurement of the mother's waist circumference and the presence of excess weight in the child. In addition, it showed that this condition was significantly associated with protein consumption by children, irregular consumption of natural fruit juice and better socioeconomic status. We also noticed a high prevalence of excess weight in children living in rural areas. These findings point to the need to implement new collective approaches in order to establish public policies related to nutrition, aimed at families living in rural areas, especially for the mother, through multi-factorial interventions considering their social contexts.

It is important to emphasize that the early onset of excess weight in children is a worrying factor, with the resulting need for educational interventions in the school environment in conjunction with the FHS of rural areas, promoting healthy life habits, such as regular practice of physical activity and a diet rich in fruits and vegetables. In addition, the incentive to cultivate gardens and orchards needs to be rescued in rural residents in order to enable greater intake of *in natura* food, thus reducing the consumption of processed foods. It is recommended to work on autonomy and to encourage an attitude to change habits even in childhood, as a way to prevent possible health problems. The importance of this study for the improvement of the Nursing praxis is highlighted, insofar as it signals to the nurses, professionals who deal directly with education in health, the need to prioritize the promotion of adequate and healthy food in every opportunity that they access children and their families, favoring the prevention of nutritional problems and contributing to the improvement of the population's quality of life.
